# Trying to Move Your Unseen Static Arm Modulates Visually-Evoked Kinesthetic Illusion

**DOI:** 10.1371/journal.pone.0080360

**Published:** 2013-11-11

**Authors:** Morgane Metral, Baptiste Blettery, Jean-Pierre Bresciani, Marion Luyat, Michel Guerraz

**Affiliations:** 1 Laboratory of Psychology and NeuroCognition, UMR 5105 CNRS – University of Savoie, Chambéry, France; 2 Department of Psychology, University of Savoie, Chambéry, France; 3 Laboratory of Functional Neurosciences and Pathologies, EA 4559, Lille, France; 4 Department of Medicine, University of Friburg, Friburg, Switzerland; Federal University of Rio de Janeiro, Brazil

## Abstract

Although kinesthesia is known to largely depend on afferent inflow, recent data suggest that central signals originating from volitional control (efferent outflow) could also be involved and interact with the former to build up a coherent percept. Evidence derives from both clinical and experimental observations where vision, which is of primary importance in kinesthesia, was systematically precluded. The purpose of the present experiment was to assess the role of volitional effort in kinesthesia when visual information is available. Participants (n=20) produced isometric contraction (10-20% of maximal voluntary force) of their right arm while their left arm, which image was reflected in a mirror, either was passively moved into flexion/extension by a motorized manipulandum, or remained static. The contraction of the right arm was either congruent with or opposite to the passive displacements of the left arm. Results revealed that in most trials, kinesthetic illusions were visually driven, and their occurrence and intensity were modulated by whether volitional effort was congruent or not with visual signals. These results confirm the impact of volitional effort in kinesthesia and demonstrate for the first time that these signals interact with visual afferents to offer a coherent and unified percept.

## Introduction

For spatial tasks, the central nervous system (CNS) tends to prioritize visual afferents over other signals in the spatial domain [1-3]. The strong weight allocated to visual inputs can mask the involvement of other afferent or efferent signals. For instance, in kinesthesia, the vibration-evoked kinesthetic illusion is abolished by the vision of a static vibrated arm in an illuminated environment [4,5]. The illusion is also suppressed if the reflection of a static arm which occupies the position of the masked vibrated arm is displayed using a mirror positioned in the midsagittal plane [6]. 

However, interaction between visual and other signals strongly depends on the sensorial context [5,7]. For instance, when the visual and proprioceptive (from muscle receptors) channels both convey signals of a moving arm, the different sensory signals can be combined in an additive way to offer a coherent percept of arm movement. In a series of experiments, Guerraz et al. [6] investigated kinesthetic illusion in both unimodal visual (mirror box paradigm) / proprioceptive stimulation (vibration paradigm) and bimodal conditions. In the unimodal visual condition, the participant’s left arm, the image of which was reflected in a mirror, was passively moved into flexion/extension. Unimodal proprioceptive stimulation consisted of vibration of either the biceps or triceps of the masked right arm. In both unimodal conditions, participants experienced kinesthetic illusions coherent with the sensory stimulation. In bimodal conditions, the visual and proprioceptive stimulations were either congruent (passive displacement of the left arm into extension / right arm biceps vibration) or opposite (eg. passive displacement of the left arm into extension / right arm triceps vibration). Interestingly, predicted illusions computed on the basis of those experienced in each unimodal condition was quite similar to and strongly correlated with the kinesthetic illusion experienced in the combined bimodal conditions, which was later confirmed by Tsuge et al. [8]. 

Clinical and experimental evidence attests that position sense and kinesthesia is not derived exclusively from afferent inflow (muscle, tactile and visual afferents [9-16]), but also from efferent outflow signals [17-19] originating from volitional efforts. Gandevia et al. [17] investigated the involvement of efferent signals (signal of motor command) in healthy participants transiently deprived of peripheral signal of position and movement of one hand combined with the paralysis of that hand (by the use of ischemia). Results showed that attempted contraction strongly shifted the position of the “phantom” hand in the direction of the voluntary effort. Therefore, similarly to phantom kinesthesia often reported by amputees [20], when it is the only available signal, the motor command strongly affects the position and movement sense. The involvement of efferent signals was later confirmed by Smith et al. [19] in more natural conditions, that is, when muscle proprioceptors are available but overt movement prevented. Although the distortion due to efferent signals was strongly reduced when muscle afferents were present (and contradictory), it was not abolished. The common feature of these experiments is that visual cues are systematically precluded. This can be easily understood considering the predilection for prioritizing visual afferents over other signals in many sensorial circumstances [1-3]. Therefore, although these results suggested a definite role of volitional effort (efferent signals) in kinesthesia, it did not reveal whether they contributed when full afferent signals, including vision, are present. Using the mirror box paradigm, i.e., a sensorial context in which vision has been shown to interact with other signals [6], the present experiment tested whether visually-induced kinesthetic illusions are modulated by volitional effort in condition of isometric contraction, either congruent or opposite to mirror reflection.

## Method

### I.1 Participants

Twenty healthy adult participants (11 females, 18 right-handed), ranging in age from 18 to 49 years (m=26.6 SD=7.8) participated in the experiment. None of the participants had a history of visual, proprioceptive or neuromuscular disease. All volunteered and provided written informed consent prior to participating in the experiment. The experiment was performed in accordance to the ethical standards laid down in the 1964 Declaration of Helsinki and approved by the local ethics committee of the University of Savoie (UDS n° 2013025).

### I.2 Material

**Figure 1:**
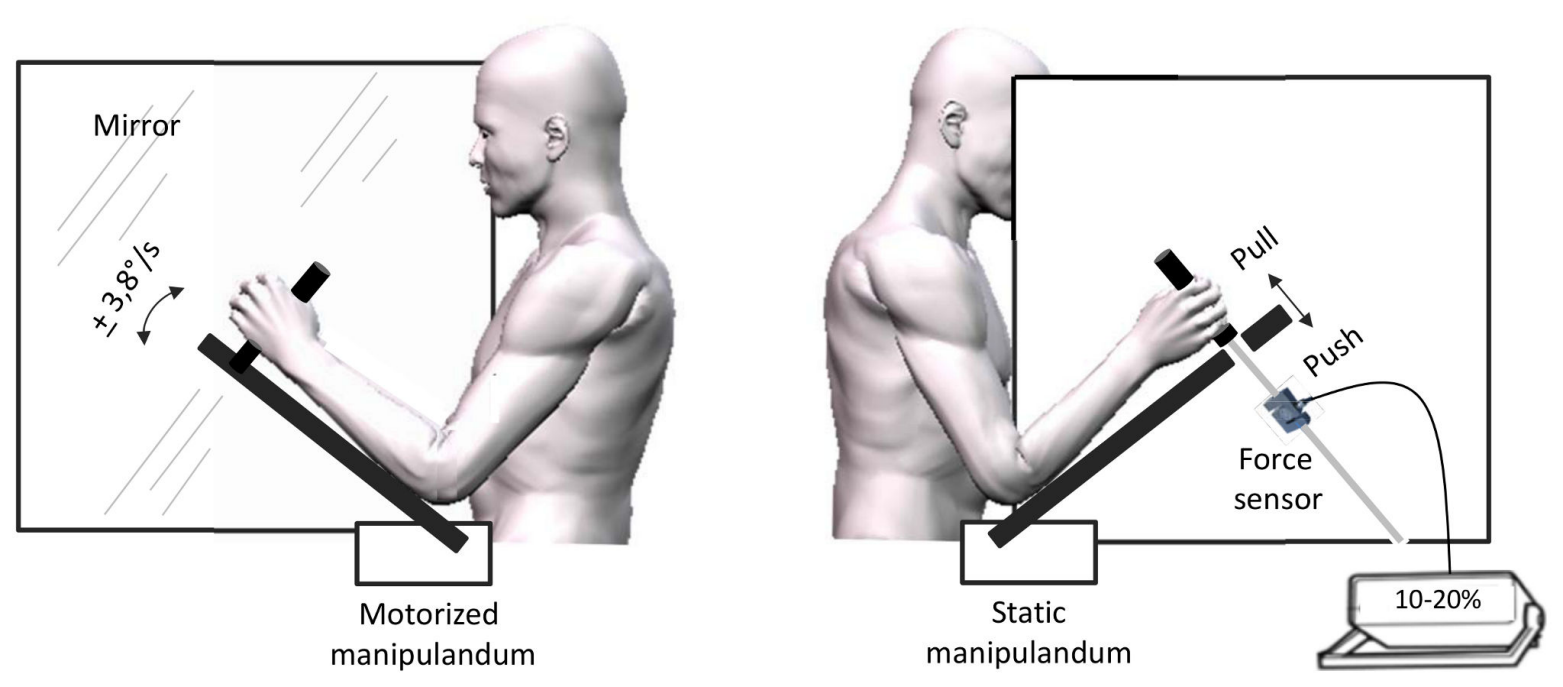
Experimental set-up. The left panel reflects the mirror side of the display with the motorized left hand. The right panel depicts the unseen static left arm (behind the mirror) on which participants exerted either pull/push effort or no effort.

Participants sat in front of a large custom built box. A mirror measuring 65 x 65 cm was positioned vertically in the middle of that box, with the reflective surface facing to the participants left arm and oriented parallel to his midsagittal axis (Fig. 1). Participants’ forearms were positioned on each side of the mirror and were supported by two manipulanda devices positioned at 45° in the starting position. The distances between the manipulanda and the mirror were adjusted so that the mirror image of the left arm coincided with the position of the right arm. The manipulanda consisted in wooden arms mounted with handles on which participants positioned their forearms and hands. The left manipulandum was motorized (Low noise DC motor) and could rotate (via a remote controller) to move participant’s left elbow into flexion or extension. Manipulandum rotation velocity was fixed at 3.8°/s. Participants’ forearms were adjusted on the manipulandum so that the axis of rotation of the motorized device precisely coincided with the participant’s elbow joint. Displacements of the manipulandum were recorded with an electromagnetic motion capture system (Polhemus Fastrak^™^, USA). A sensor was positioned on the device so that continuous signals (collected with a sampling frequency of 60 Hz) of the angles of the manipulandum were provided. 

The right manipulandum was fixed. The handle was fixed to a tension and compression sensor force (TME, F501 TC) and could not move even in condition of maximal voluntary force. This sensor was connected to a portable signal conditioner displaying the applied force in daN. This digital display was monitored online by the experimenter to control the force deployed by the participant (see procedure) but was not visible to the participant. EMG activities of the biceps and triceps brachii of the right arm were also recorded and analyzed offline to check for the pattern of EMG activity in the pull, push and rest conditions. EMG was recorded via surface electrodes placed with a 3cm inter-electrode distance longitudinally over the bellies of the muscles. A reference electrode was attached to a body area remote from the studied muscles. EMG data were sampled at 500 Hz with a Biopac device (Biopac systems Inc). Offline EMG analysis confirmed that participants exerted a limited force in the push and pull conditions as requested (see procedure); the mean integrated EMG of agonist muscles in the push (triceps) and pull (biceps) conditions were 12.4 % and 9 % of the maximal voluntary force (MVF) respectively. The mean integrated EMG activity did not exceed 2.5 % of MVF in the two antagonist muscles in the rest condition. 

### I.3 Procedure

Throughout the experiment, participants were required to look at the reflection of their left arm through the mirror orientated parallel to their midsagittal axis. Vision of the right arm was prevented. Both the right and left forearms were positioned at an angle of 45° to the horizontal prior to trial onset. Prior to experimental trials, participants were required to exert maximum force into flexion (Pull) or extension (Push) with their right arm against the unmovable handle/manipulandum. Based on this maximal effort into flexion and extension, they were trained to practice “Push” and “Pull” efforts with a force of an estimated 10-20% maximum effort. In experimental trials, following a baseline epoch of ~10s without any arm displacement, the left forearm could be passively moved for 10s at a constant velocity of 3.8°/s. Participants were instructed not to resist to this passive displacement. Three “Mirror movement” conditions were carried out: the left forearm, and therefore its reflection through the mirror, was passively moved either in “Flexion” (1), “Extension” (2), or remained “Static” (3). These three Mirror conditions were paired with three “*Effort*” conditions in a within subjects design: In the “Push” and “Pull” conditions, participants were required to either extend (“Push”) or flex (“Pull”) their right arm, to reach an effort of ~10-20% maximum. They exerted this effort on verbal instruction (start and stop) from the onset to the offset of left arm displacement. In the third condition, participants were required not to exert any force into flexion or extension (“Rest”). Each condition was repeated 3 times in pseudo-random order for a total of 27 trials per participant. Participants moved actively and synchronously their two arms into flexion-extension before each trial. This allowed the two arms to have a similar immediate history of contraction and length changes before trials [21,22]. 

### I.4 Measure of kinesthetic illusion

#### Subjective report

During the trial, participants were required to verbalize when they felt their right arm started moving (illusory displacement). At the end of the trial, they were required to rank the direction (flexion or extension) and speed of the felt displacement (illusion) of the right arm on a subjective scale ranging from 0 to 20 with steps of one. Zero corresponded to the absence of felt displacement of the referenced arm, ten being a felt displacement which velocity was equal to that of the passively moved left forearm. Twenty corresponded to a felt displacement which velocity was twice as important. Prior to the experiment, participants experienced trials with passive displacement of the left forearm to become familiar with subjective rating. This subjective rating was evaluated in a recent publication and it was shown to strongly correlate with judgment of position and velocity reproduced with the foot (see 6). Since in the present experiment participants were already engaged in a motor action (voluntary effort with the right forearm), foot adjustment was not considered. Illusions were quoted as positive when the felt displacement was in the direction of a flexion and negative when the felt displacement was in the direction of an extension.

### I.5 Statistics

The χ^2^-test was used to compare the percentage of illusion occurrence in the different experimental conditions. When the left arm was static (“*Mirror-*static”), kinesthetic illusion occurred extremely rarely in the “Push” and “Pull” *effort* conditions (illusion occurred in only 4 out of 120 trials ~3% ) and did never occur in the “Rest” *effort* condition. Therefore, those experimental conditions were not included in the statistical analyses. Accordingly, illusion intensity were analyzed using a 2x3 [“Mirror-movement” (Extension – Flexion) * “Effort” (Pull, Rest, Push)] repeated measures ANOVAs (within subjects design). Generalized eta square (η^2^_G_) was reported (See 23 for recommendation). The reported values are Huynh-Feldt corrected and post hoc tests were performed using Holm correction for multiple comparisons. *S*ignificance was set at 0.05. 

## Results

### Occurrence of kinesthetic illusion

#### Kinesthetic illusion in the “Mirror-static” condition

As mentioned above, kinesthetic illusion occurred only on very rare occasions when the left arm (and its reflection through the mirror) was static, whether the *effort* of the right arm was “Pull” (1.6%), “Push” (1.6%) or “Rest” (0%). 

#### Visually-induced kinesthetic illusion in the “rest” effort condition

Reflection of the passively moving left arm through the mirror often evoked kinesthetic illusions of right arm displacement in the same direction, that is, “mirror illusion”. When participants were required to relax their right arm (“Rest”), mirror illusion occurred in 86% (52/60 trials), whereas they occurred in 83% (50/60 trials) of the trials when the arm was moved into flexion (mirror-flexion) and extension (mirror-extension), respectively. The difference did not reach significance (χ^2^ (1) <1 p>0.05). No illusion occurred in 12% and 15% of the trials in conditions of mirror-flexion and extension, respectively. Very few kinesthetic illusions which direction was opposite to visual stimulation were reported when the arm was moved either into flexion (mirror-flexion: 1.6%; 1/60 trials) or extension (mirror-extension: 1.6%; 1/60 trials). 

#### Visually induced illusion in the “Pull” and “Push” effort conditions

**Figure 2:**
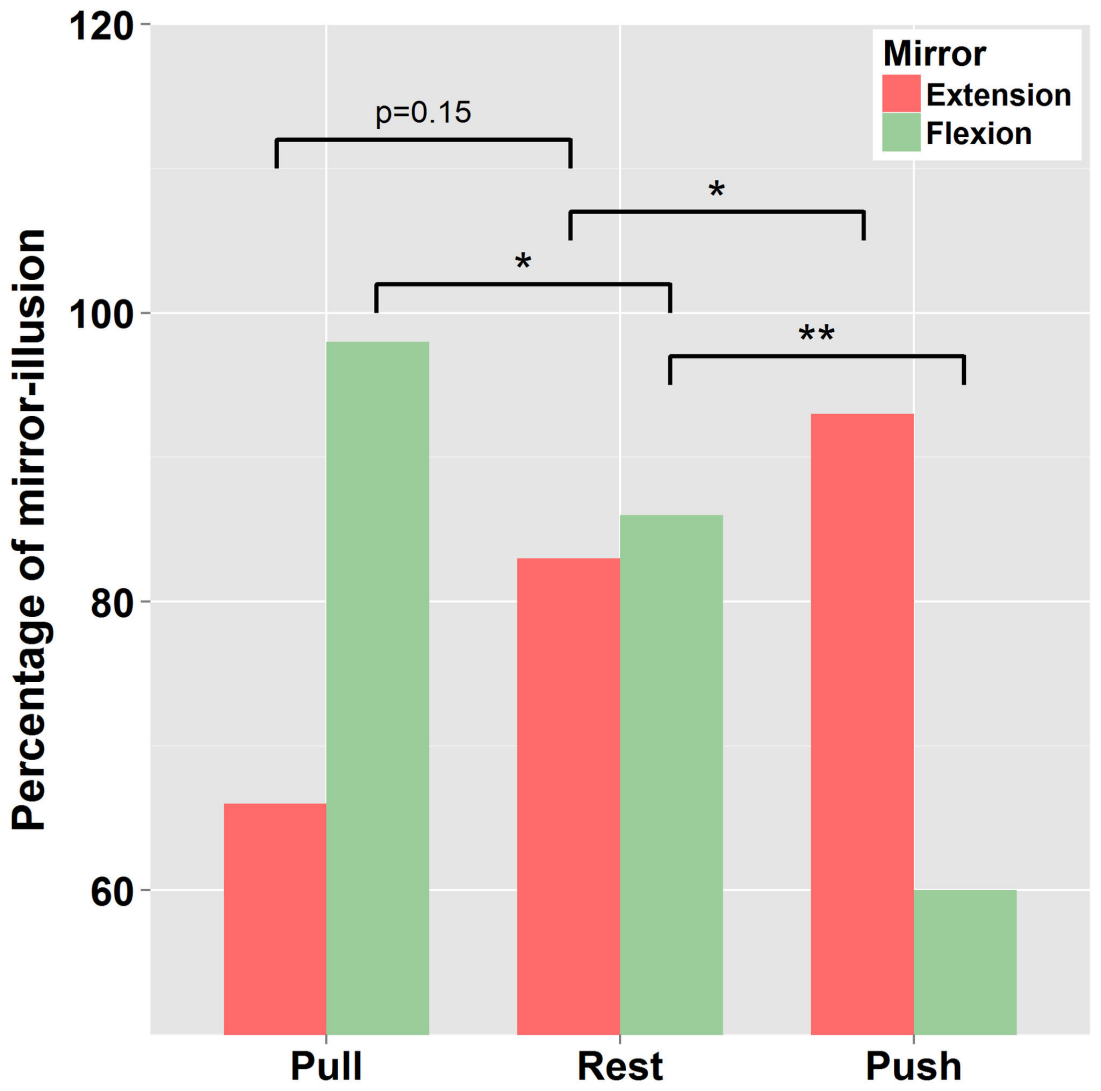
Occurrence of visually-induced kinesthetic illusion: Percentage of illusion occurrence with the passive displacement of the left arm in condition of mirror-flexion / mirror-extension in the three effort conditions (pull, rest and push). Asterisks indicate significance (* = p<.05, ** = p<.01).

As can be seen in Figure 2, the occurrence of visually induced kinesthetic illusion (mirror illusion) varied with Effort conditions. As compared to “Rest” (86%), when the left arm was moved passively into flexion (mirror-flexion), illusion occurrence decreased when participants were required to make an effort into extension (“Push” [opposite to mirror movement] : 60 %, χ^2^ (1)=9.6 p<0.01) and increased when required to make an effort into flexion (“Pull” [congruent with mirror movement]: 98%, χ^2^ (1)=4.3 p<0.05). Similarly, when the left arm was moved passively into extension (mirror-extension), the occurrence of mirror illusion tended to increase when participants were required to make an effort into extension (“Push” [congruent with mirror movement]: 93.3 %, χ^2^ (1)=2.2 p=0.15, “Rest” = 83%) and decrease when required to make an effort into flexion (“Pull” [opposite to mirror movement]: 66%, χ^2^ (1)=3.6 p=0.05). To sum up, visually driven illusion (mirror illusion) occurred in a high percentage of trials. In addition, the occurrence of mirror illusion increased when the effort was congruent with the passive displacement of the left arm (and its reflection through the mirror), and decreased when incongruent with this passive displacement. 

In a few trials, participant reported kinesthetic illusions in the opposite direction to the passively moving left arm through the mirror. These kinesthetic illusions that were in the direction of effort, occurred only when effort was opposite to visual stimulation (“Pull – mirror-flexion” 8.33%, 5/60 & “Push – mirror-extension” 10%, 6/60). 

Finally the highest percentage of trials in which no illusion was reported occurred when mirror movement and effort conditions were not congruent, that is in the “pull – mirror- extension” (23.3 %, 14/60 trials) and “push - mirror-flexion” (31.6%, 19/60 trials) conditions. 

The occurrence of kinesthetic illusion (expressed in terms of frequency) for each experimental condition was reported in table 1. We also report in table 1 the frequency of participants that experienced either systematically or at least twice a kinesthetic illusion in the three trials per experimental condition. 

**Table 1:** Upper table: Frequency of participants that experienced kinesthetic illusion in 3 or 2 trials over the 3 trials per experimental condition.

Frequency of participants that experienced illusion 2 or 3 times out of 3 trials			
	Pull	Rest	Push
Extension	0.75	0.9	1
Static	0.1	0	0.1
Flexion	1	0.9	0.7
Frequency of illusion occurrence			
Extension	66.6	83.3	0.933
Static	0.016	0	0.016
Flexion	0.983	0.86	0.6
Mean intensity of kinesthetic illusion			
Extension	-3.7 (1.4)	-4.9 (1.25)	-6.2 (1.23)
Static	0.16 (0.23)	0 (NA)	-0.01 (0.2)
Flexion	6.3 (1.24)	5.7 (1.23)	2.8 (1.6)

Middle table: Frequency of kinesthetic illusion in the different experimental conditions. Lower table: Mean intensity of kinesthetic illusion in the different experimental condition. Illusions were quoted as positive when the felt displacement was in the direction of a flexion and negative when the felt displacement was in the direction of an extension.

### Ratings of kinesthetic illusion (speed)

**Figure 3:**
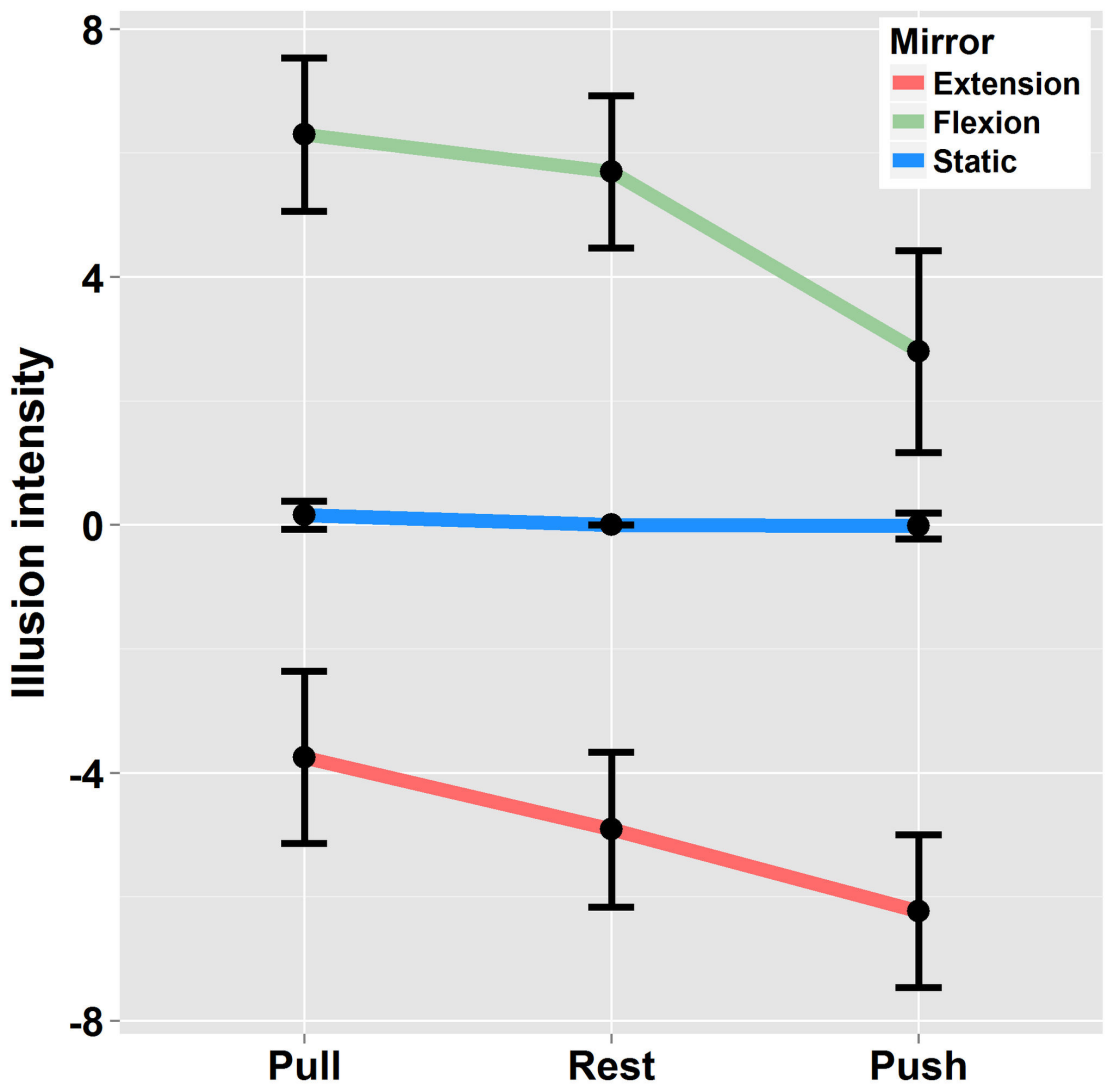
Illusion intensity: Ratings in condition of Mirror-flexion / Mirror-extension and static left arm in the three Effort conditions. Illusions were quoted as positive when the felt displacement was in the direction of a flexion and negative when the felt displacement was in the direction of an extension. Errors bars are interval confidence.

The ANOVA revealed a significant main effect of “Mirror-movement” (F(1,19)= 99 p<.01 η^2^_G_ =.73). Indeed, reflection of the passively moving left arm through the mirror evoked illusory extension of the right forearm in the “Mirror-extension” condition (quoted positively) and illusory flexion in the “Mirror-flexion” condition (quoted negatively). Although vision largely dominated the percept, the illusory displacement of the right arm was modulated by effort conditions (F(2,38)=16.9 p<.01 η^2^_G_ =.15) as depicted in Figure 3. As compared to the “Rest” condition, the right arm was perceived to move faster when the reflection of the moving left arm was congruent with the Effort produced in the right arm (Mirror-extension / Push and Mirror-flexion / Pull). In contrast, it was perceived as moving slower when the reflection of the moving left arm was incongruent with the Effort produced in the right arm (Mirror-extension / Pull and Mirror-flexion / Push). Post-hoc analysis showed that the mean kinesthetic illusion in the “Pull” condition differed significantly from the ‘”Push” when the left arm was moved either into flexion or extension (p<.05). The difference between the “Pull” or “Push” conditions and the “Rest” condition did not systematically reach significance (Mirror-extension: Pull vs Rest p>0.5 – Push vs Rest p>0.05; Mirror-flexion: Pull vs Rest p>0.5 - Push vs Rest p<0.01). Finally, no interaction occurred between the “Mirror-Movement” and “Effort” factors (F(2,38)=1.14 p>.05 η^2^_G_ =.01). 

**Figure 4:**
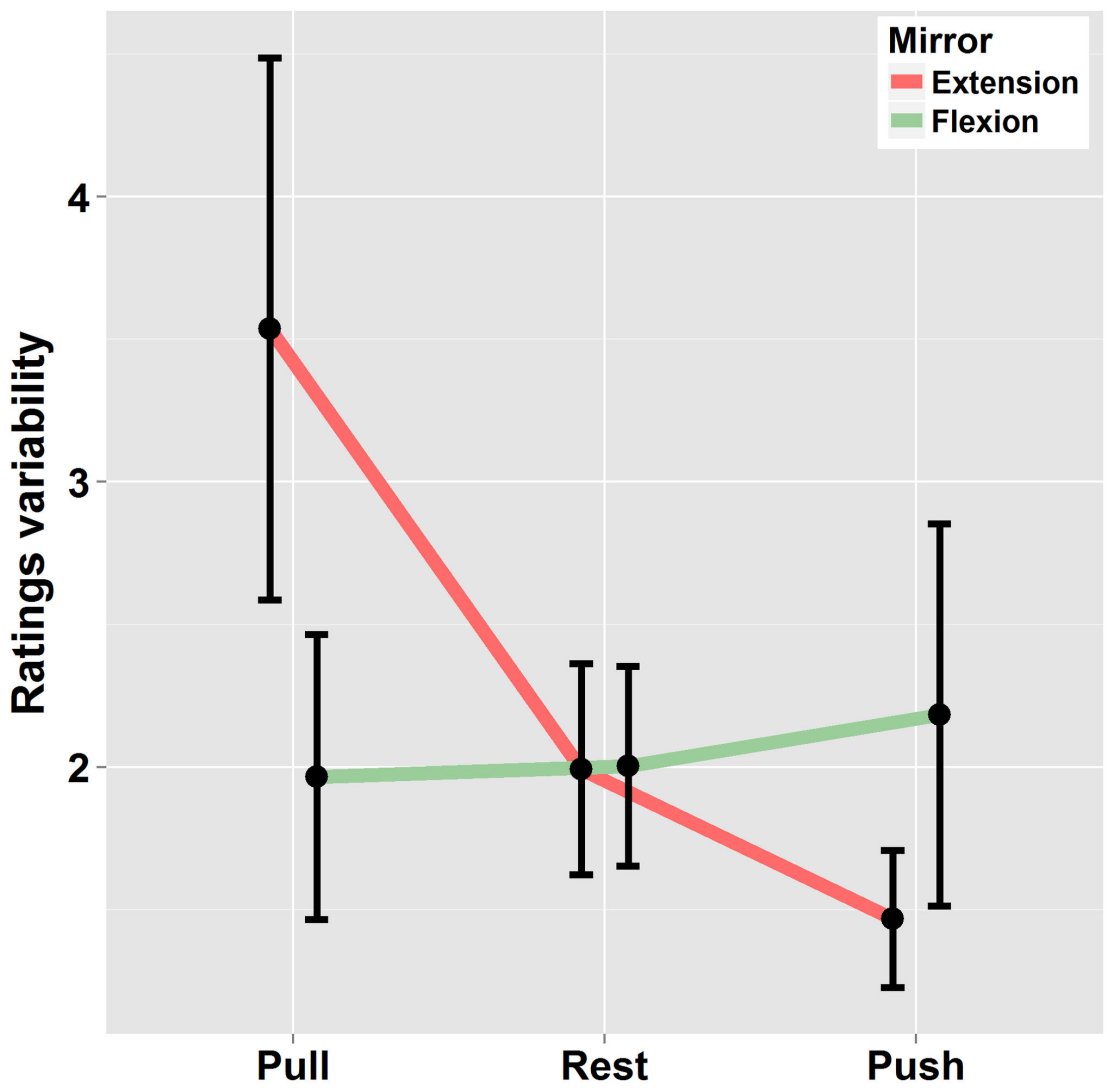
Standard deviation of ratings in condition of Mirror-flexion / Mirror-extension and static left arm in the three Effort conditions. Errors bars are interval confidence.

In a supplementary analysis, within-subject rating variability was analyzed. For that purpose, the standard deviation was calculated for each participant in the different experimental conditions (on the basis of three trials per experimental condition). Data were then submitted to an ANOVA using a 2x3 [“Mirror-movement” (Extension – Flexion) * “Effort” (Pull, Rest, Push)] repeated measures. As can be seen in Figure 4, rating’s variability increased when visual signals (reflection of left arm displacement) were opposite to volitional effort of the right arm and decreased when congruent. This is confirmed by the significant interaction between “Mirror-movement” and “effort” (F(2,38) = 4.4 p<.05 η^2^_G_ =.04). This effect was more pronounced when the mirror extension was concerned. 

## Discussion

In the present experiment, participants frequently reported illusory displacements of their right unseen forearm when looking at the mirror reflection of their passively moved left forearm (mirror illusion). This attests of the large involvement of visual afferents in kinesthesia and confirms previous observation [6]. Importantly, volitional efforts to move the right forearm while movement was prevented (isometric effort) affected both the occurrence and intensity of the mirror illusion. 

Gandevia et al. [17] and Smith et al. [19] have elegantly demonstrated the involvement of volitional efforts in position sense and kinesthesia. Theses authors showed that although the magnitude of the bias in position sense attributed to the motor command is reduced in condition of intact proprioceptive afferents, it does not completely vanish (when compared to complete paralysis and anesthesia, e.g., due to ischemia). In these studies, the use of visual information was systematically precluded. In the present experiment, we assessed the contribution of volitional effort in kinesthesia when visual information is available. Our results show that when visual signals and volitional control were congruent, the occurrence of mirror illusion and its intensity increased as compared to conditions presenting the same visual information but in which no volitional effort was produced. When visual information and volitional effort were opposite, the occurrence and intensity of the illusion decreased. These results indicate that the CNS takes advantage of *all* available sources of information including motor signals in order to build a coherent percept of arm movement. 

The way volitional control participates to kinesthetic perception is not straightforward. It has often been proposed to be useful to separate self-generated movements from those produced by an external agency [24,25] and therefore alleviate sensory ambiguities. Here, volitional control modulated both the occurrence and intensity of the mirror illusion. In addition, when volitional control was congruent with visual information, the variability of the responses was reduced (rating’s variability measured from one trial to another in a given experimental condition). This suggests that in condition of sensorial mismatch between visual and muscle proprioceptive signals, as in the mirror paradigm (visual signals of a moving arm & muscle proprioceptive signals of a static arm), the motor command either reinforce the trustworthiness of visual information when congruent, or weaken it when opposite. Motor signals could therefore be integrated with sensory afferents and contribute to the final percept. Interestingly, in our experiment, the illusion was nearly systematically driven by the visual input. This suggests that the weight allocated to visual information was higher than that allocated to motor signals. This would be consistent with the high weight usually allocated to visual signals in spatial tasks [3], and would make much sense considering that motor signals are inherently ambiguous when kinesthetic perception is concerned. Indeed, motor commands do not systematically lead to overt movements and if so, the overt movement might well differ from the expected one as a consequence of external physical constraints for instance.

When the mirror reflected a static hand, isometric contractions did not trigger any illusion, suggesting that in this condition, the motor command was mostly ignored. As reported initially by Goodwin et al [4], vision of a static vibrated arm in an illuminated environment prevents vibration-evoked illusion to occur. Similarly, Guerraz et al [6] reported that the vibration-evoked kinesthetic illusion vanishes when participants saw the reflection of a static arm in lieu of the masked vibrated arm. Therefore, when the visual channel conveys signals of a static hand, at least when the visual context is sufficiently rich [5], the visual signal dominates the percept and both proprioceptive afferents and efferent signals can be largely ignored. The reasons of such dominance of visual signals likely stems from the high acuity of the visual system to detect motion (low absolute threshold of visual motion detection). In that respect, the CNS likely gives priority to such highly reliable signals attesting of immobility of the arm (static arm condition) than to more labile proprioceptive signals [26] or inherently ambiguous motor signals. 

It is difficult to allocate with certainty the significant effect of isometric contraction in our experiment to either the central command or its peripheral consequences. Isometric contraction is accompanied with i. shortening, though limited, of muscle fibers [27], ii. possible recruitment of spindle endings [28], iii. increased tension applied on the golgi organ tendons and iv. cutaneous pressure in the hand. Although the involvement of those afferent signals cannot be fully discarded, expectation based on the physiological properties of these afferents does not fit with our results. In condition of isometric contraction, changes in cutaneous pressure in the palm likely signal resistance to movement rather than movement per se what contrasts with the increased illusory displacement when the motor and visual signals were congruent. The involvement of spindle endings appears also unlikely. Indeed, Burke et al [28] reported that in condition of isometric contraction, spindle endings activity may occur as a consequence of fusimotor drive (if strength is superior to 1.5 Nm) but only in the homonymous muscle endings [28,29]. Although spindle activity is of primary importance in kinesthesia, increased activity is mainly associated with muscle lengthening. If involved, it would rather have reinforced the kinesthetic illusion when volitional effort was not congruent with visual inputs, and *vice et versa*, what is opposite to our results. Finally, tendon organs also increase their discharge during contraction [29]. Emerging data suggest that those organs contribute to proprioception but more precisely to the senses of effort and heaviness (see 30 for a review) rather than position and movement. 

The present results tend to confirm the contribution of efferent signals to kinesthesia. Importantly, they show for the first time that when visual information is available, volitional effort signals interact with visual afferents to build a coherent percept. This interaction might be of interest in clinical practice, for instance with patients suffering from phantom pain. Indeed, volitional control could be combined with the classical mirror therapy, suggested to alleviate pain symptoms [31]. It may facilitate reanimation of lost phantom kinesthesia or help opening of the phantom hand in case of clenching spasms for instance. 
